# The challenges of designing a benchmark strategy for bioinformatics pipelines in the identification of antimicrobial resistance determinants using next generation sequencing technologies

**DOI:** 10.12688/f1000research.14509.2

**Published:** 2018-12-07

**Authors:** Alexandre Angers-Loustau, Mauro Petrillo, Johan Bengtsson-Palme, Thomas Berendonk, Burton Blais, Kok-Gan Chan, Teresa M. Coque, Paul Hammer, Stefanie Heß, Dafni M. Kagkli, Carsten Krumbiegel, Val F. Lanza, Jean-Yves Madec, Thierry Naas, Justin O'Grady, Valentina Paracchini, John W.A. Rossen, Etienne Ruppé, Jessica Vamathevan, Vittorio Venturi, Guy Van den Eede

**Affiliations:** 1 European Commission Joint Research Centre, Ispra, 21027, Italy; 2 Department of Infectious Diseases, Institute of Biomedicine,The Sahlgrenska Academy, University of Gothenburg, Gothenburg, SE-413 46, Sweden; 3 Centre for Antibiotic Resistance research (CARe) , University of Gothenburg, SE-413 46, Gothenburg, Sweden; 4 Institute for Hydrobiology, Technische Universität Dresden, Dresden, 01307, Germany; 5 Canadian Food Inspection Agency, Ottawa Laboratory (Carling), Ottawa, ON, K1A 0Y9 , Canada; 6 International Genome Centre, Jiangsu University, Zhenjiang, China; 7 Division of Genetics and Molecular Biology, Institute of Biological Sciences, University of Malaya, Kuala Lumpur, 50603, Malaysia; 8 Departamento de Microbiología, Hospital Universitario Ramón y Cajal, Instituto Ramón y Cajal de Investigación Sanitaria (IRYCIS), Madrid, 28034, Spain; 9 BIOMES.world, c/o Technische Hochschule Wildau, Wildau, 15745, Germany; 10 Unité Antibiorésistance et Virulence Bactériennes, ANSES Site de Lyon, Lyon, F-69364 , France; 11 Service de Bactériologie-Hygiène, Hôpital de Bicêtre, Le Kremlin-Bicêtre, F-94275, France; 12 Norwich Medical School, University of East Anglia, Norwich, NR4 7TJ , UK; 13 Department of Medical Microbiology, University Medical Center Groningen, University of Groningen, Groningen, 9713 GZ , The Netherlands; 14 Laboratoire de Bactériologie, Hôpital Bichat, INSERM, IAME, UMR 1137, Université Paris Diderot, Paris, F-75018, France; 15 European Molecular Biology Laboratory, European Bioinformatics Institute (EMBL-EBI), Hinxton, CB10 1SD, UK; 16 International Centre for Genetic Engineering and Biotechnology (ICGEB), Trieste, 34149, Italy; 17 European Commission Joint Research Centre, Geel, B-2440, Belgium

**Keywords:** Antimicrobial resistance, bioinformatics, next-generation sequencing, benchmarking

## Abstract

Next-Generation Sequencing (NGS) technologies are expected to play a crucial role in the surveillance of infectious diseases, with their unprecedented capabilities for the characterisation of genetic information underlying the virulence and antimicrobial resistance (AMR) properties of microorganisms.  In the implementation of any novel technology for regulatory purposes, important considerations such as harmonisation, validation and quality assurance need to be addressed.  NGS technologies pose unique challenges in these regards, in part due to their reliance on bioinformatics for the processing and proper interpretation of the data produced.  Well-designed benchmark resources are thus needed to evaluate, validate and ensure continued quality control over the bioinformatics component of the process.  This concept was explored as part of a workshop on "Next-generation sequencing technologies and antimicrobial resistance" held October 4-5 2017.   Challenges involved in the development of such a benchmark resource, with a specific focus on identifying the molecular determinants of AMR, were identified. For each of the challenges, sets of unsolved questions that will need to be tackled for them to be properly addressed were compiled. These take into consideration the requirement for monitoring of AMR bacteria in humans, animals, food and the environment, which is aligned with the principles of a “One Health” approach.

## 1. Introduction

Next-Generation Sequencing (NGS) technologies are increasingly regarded as an essential tool in modern regulatory frameworks. Monitoring schemes that rely on the characterisation of genetic information will gain considerably by utilising these technologies. Their importance for infectious diseases surveillance was highlighted by “The Review on Antimicrobial Resistance” in 2014, which stated that “advances in genetics, genomics and computer science will likely change the way that infections and new types of resistance are diagnosed, detected and reported worldwide, so that we can fight back faster when bacteria evolve to resist drugs” ^1^.

This interest can be observed in the rapid expansion in recent years of whole-genome sequencing capacities in national public health infectious diseases surveillance laboratories, as recently reported in a European survey by the European Centre for Disease Prevention and Control (ECDC) ^2^. Antimicrobial resistance (AMR), i.e. the ability of a microorganism to resist the action of an antimicrobial agent, is of particular importance in this surveillance program. Its observed rise places heavy burdens on healthcare systems, leading to prolonged treatment times, higher mortality and high economic impacts (see 3). In March 2017, the Joint Research Centre organised a meeting in order to better understand the state-of-the-art of the application of NGS technologies in the fight against AMR ^4^. Although it is clear that the uses of NGS vary according to the specific need (e.g. to guide clinical intervention or to evaluate the environmental and human health risks of AMR genetic determinants), these discussions highlighted overlaps in the needs and the challenges of implementing NGS for the monitoring of AMR in humans, animals, food and the environment. Some of these were also highlighted in previous workshops organized by the European Food Safety Authority (EFSA) and the ECDC ^5, 6^.

**Figure 1.**
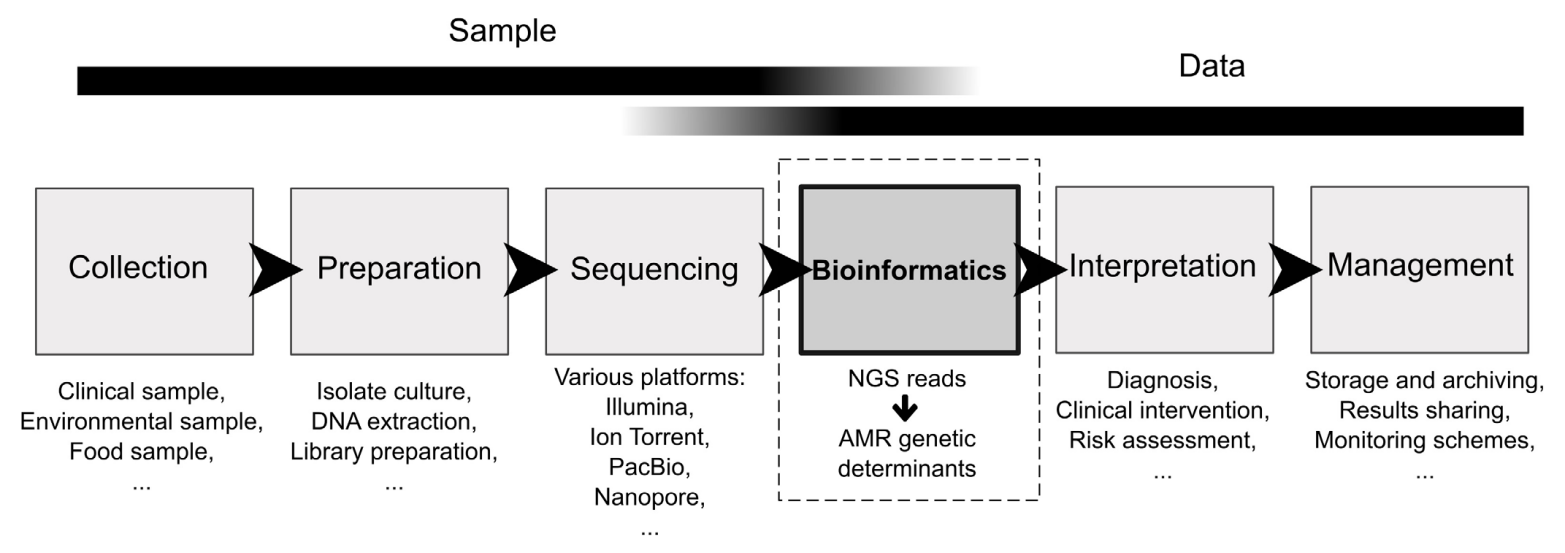
Overview of the different steps involved in the use of Next-Generation Sequencing technologies for the detection and monitoring of antimicrobial resistance. The benchmark strategy discussed in the current article focuses on the bioinformatics steps, the pipeline converting the output of the sequencing experiment into a list of identified antimicrobial resistance genetic determinants (dashed rectangle).

A full regulatory implementation of NGS technologies to monitor AMR will need to address many standardisation challenges throughout the process, which broadly includes sample preparation and DNA extraction, library preparation for sequencing, the use of an NGS instrument for generating the sequences, the bioinformatics analysis, and interpretation and reporting of results (see Figure 1). Focusing on the bioinformatics step, an important shared challenge is the need to correctly and reliably identify the known genomic determinants of AMR from a set of NGS reads produced from sequencing a sample. The ECDC study reported the requirement for sufficient bioinformatics expertise as one of the important hurdles to a more general implementation of NGS for routine testing ^2^. This observation has also been made in recent case studies and reviews ^7– 11^.

By contrast, within the scientific research community the recent literature reflects widespread enthusiasm for the application of NGS approaches to the determination of AMR characteristics in bacteria. For the bioinformatics steps, many useful strategies have been published. These are, however, very varied in the approaches and resources they use. Some start with sequencing reads produced by the Illumina ^12, 13^, Ion Torrent ^14^, PacBio ^15^ or Nanopore ^16^ platforms, just to give a few examples. To predict the resistance profile, interesting results were reported with very different strategies, including k-mer analysis of the reads ^17^, sequence comparisons of individual reads to databases ^12, 16^, first assembling the reads into contigs using various software packages ^9, 18^ and building and comparing de Bruijn graphs of the sequenced sample reads and the reference database ^19^. The reference set of genetic determinants of AMR used by the bioinformatics pipelines also varied, including databases such as ARGANNOT ^20^, CARD ^16^, ResFinder ^9^, Resqu ^12^, ARDB ^21^, custom-generated from Genbank sequences ^18, 22^ or combinations of these ^14^. Interestingly, the choice of the database was shown to greatly influence the interpretation of risk associated with AMR in public health ^23, 24^. Even individual steps, such as mapping sequenced reads to a reference, can be done with different tools, each carrying their own compromises (see 25– 28).

This complex - and dynamic - reality poses a challenge for the implementation of bioinformatics pipelines in regulatory settings, where the demonstration of reliability and reproducibility is crucial (see also 11, 29). Harmonisation approaches must face the variability described above in terms of technologies, strategies, and software used, each with their demonstrated success, limitations and caveats. A further factor influencing the complexity of applying a given bioinformatics pipeline is that new versions of the individual tools that perform tasks such as quality-checking, trimming or assembling the reads, are constantly being released, which may have unanticipated impacts on pipeline performance. Ready-made and/or commercially available solutions that aim to facilitate the implementation of a NGS-based pipeline by lowering the technical skill required (see, for example, 30, 31) face the attendant “black-box” issues when proposed for regulatory purposes.

In response to this complex state-of-the-art and the fast-moving environment in which these technologies are developing, efforts for the standardisation and development of best practices have avoided the prescription of restrictive guidelines, methods or technologies in favour of a more flexible approach emphasising quality metrics and fitness-for-purpose ^32, 33^. For bioinformatics pipelines, the development of benchmark resources would play an important role in validating specific bioinformatics strategies and workflows, testing any update to the software underlying an established pipeline or allowing proficiency testing of individual laboratories ^33– 35^. These resources would need to include a set of inputs for the bioinformatics pipelines (“ *in silico* reference materials”) linked to a “correct” expected output, as well as consideration for the minimum performance requirements to be met by the pipelines. Different initiatives are ongoing to develop these benchmarking resources including, for example, the Critical Assessment of Metagenome Interpretation (CAMI) project for the evaluation methods for metagenome analysis ^36^.

On the 5 ^th^ of October 2017, the Joint Research Centre invited experts in the field of AMR monitoring in order to discuss the challenges involved in the development of such a benchmark strategy, for the specific purpose of evaluating the bioinformatics pipelines that transform a set of NGS reads to a characterised AMR profile. The experts were invited based on their recent publications on the use of NGS to detect the genetic determinants of AMR in diverse fields: human and veterinary health, the food chain and the environment. The conclusions of these discussions are summarised in Table 1, and discussed in this document.

**Table 1.** Summary of the challenges identified in the generation of benchmark datasets for the purpose of evaluating the bioinformatics pipelines that process a set of NGS reads into a characterised AMR profile. See text for details.

Section	Challenges	Questions to be addressed
**2.1**	Nature of the benchmark datasets - NGS platforms	How should a benchmark strategy handle the current and expanding universe of NGS platforms? What should be the quality profile (in terms of read length, error rate, etc.) of *in silico* reference materials? Should different sets of reference materials be produced for each platform? In that case, how to ensure no bias is introduced in the process?
	Nature of the benchmark datasets - datasets origin	Should *in silico* reference material be composed of the output of real experiments, or simulated read sets? If a combination is used, what is the optimal ratio? How is it possible to ensure that the simulated output has been simulated “correctly”? For real experiments datasets, how to avoid the presence of sensitive information?
	Nature of the benchmark datasets - quality metrics	Regarding the quality metrics in the benchmark datasets (e.g. error rate, read quality), should these values be fixed for all datasets, or fall within specific ranges? How wide can/should these ranges be?
**2.2**	Samples composition - resistance mechanisms	How should the benchmark manage the different mechanisms by which bacteria acquire resistance? What is the set of resistance genes/mechanisms that need to be included in the benchmark? How should this set be agreed upon?
	Samples composition - bacterial species	Should different sample types (isolated clones, environmental samples, …) be included in the same benchmark? Is a correct representation of different bacterial species (host genomes) important?
**2.3**	Evaluation of pipeline performance - dataset characterisation	How can the “true” value of the samples, against which the pipelines will be evaluated, be guaranteed? What is needed to demonstrate that the original sample has been correctly characterised, in case real experiments are used?
	Evaluation of pipeline performance - performance thresholds	How should the target performance thresholds (e.g. specificity, sensitivity, accuracy, …) for the benchmark suite be set? What is the impact of these targets on the required size of the sample set?
**2.4**	Generation, distribution and update of the benchmark - future proofing	How can the benchmark stay relevant when new resistance mechanisms are regularly characterised? How is the continued quality of the benchmark dataset ensured?
	Generation, distribution and update of the benchmark - ownership	Who should generate the benchmark resource? How can it be efficiently shared?

## 2. The challenges

Although some of the challenges considered reflect the reality of NGS technologies in general, efforts were made to highlight the issues that are specific to the identification of AMR determinants. Broadly, the challenges can be grouped in different, often overlapping categories.

### 2.1. Nature of the benchmark datasets

***How should a benchmark strategy handle the current and expanding universe of NGS platforms? What should be the quality profile (in terms of read length, error rate, etc.) of “in silico reference materials”? Should different sets of reference materials be produced for each platform? In that case, how to ensure no bias is introduced in the process?***

As described in the Introduction, different NGS technology platforms exist for the generation of sequence data serving as inputs for the bioinformatics processes used in the analysis of AMR determinants. Moreover, the technology continues to evolve rapidly with the advent of what is now termed “third generation sequencing” methods that can read the nucleotide sequences at the level of single molecules ^37^. Focusing on validating the technology or the instrument itself is therefore not a useful approach to ensure the reliability of the bioinformatics steps, since it can reasonably be expected that sequencing technologies and protocols will undergo many changes over the coming years. Section 862.2265 of the FDA↩s Code of Federal Regulations Title 21 ^38^ regulates the general use of NGS instruments for clinical use; even when, in this context, devices are cleared as Class II exempt ^1^, laboratories using these instruments must still establish a bioinformatics pipeline for their intended use ^39^. Thus, an effective benchmark strategy will be independent of existing and upcoming NGS technologies, while avoiding any bias that would favour one technology to the detriment of others.

The proprietary nature of the different raw data outputs produced by the various technologies may not be a primary consideration for present purposes since standard file formats exist that can store raw reads and the associated metadata (ex. QC metrics) produced by the different sequencers. These include FASTQ ^40^ and BAM ^41^, and they have been successfully used in laboratory proficiency testing ^34, 35, 42^. More recent platforms produce outputs using the HDF5 standard or variants of it; conversion into FASTQ would require an additional computational step, using one of the available tools. However, all platforms (as well as sequencer models and versions within each platform) have differences in the profile and amount of raw reads produced, with variations in their number, length, error rates, error types, etc. ^43, 44^. Attempting to create a single set of *in silico* reference materials would either introduce a bias towards a specific platform and/or create a dataset which is not representative. Creating individual sets of reads would increase the work (with no end in sight as platforms appear or evolve) and require careful consideration to avoid, once again, bias.

All this highlights a clear challenge, which is how to address both the evolution of the platforms, differences amongst instruments and run-to-run variabilities, in view of the need for benchmark datasets serving as the basis for the validation and harmonisation of NGS approaches in clinical and/or regulatory frameworks.

***Should in silico reference material be composed of the output of real experiments, or simulated read sets? If a combination is used, what is the optimal ratio? How is it possible to ensure that the simulated output has been simulated “correctly”? For real experiment datasets, how to avoid the presence of sensitive information?***

The core component of a benchmark resource is, by definition, a set of inputs representative of what the benchmarked bioinformatics pipeline is expected to receive in normal, real-life use. A logical source for this dataset, then, is the actual output of laboratory sequencing experiments ^17, 34^. However, using data generated by real experiments assumes a high level of quality that will need to somehow be assessed and demonstrated. These experiments will need to be properly characterised in terms of the “true” conclusions the benchmarked pipeline is expected to reach. In addition, although there can be actions taken to ensure that most of the host DNA is filtered from the dataset, real metagenomics experiments from a human source could lead to privacy problems, while samples from food should ensure the absence of information on patented genetically modified food potentially present in the sample ^8, 45^. Careful filtering against a standard “exclusion database”, or other adequate strategies, may be necessary to solve this issue. For example, de-identification processes for human DNA sequences have been proposed for clinical datasets. However, the impact of applying tools that modify in any way the reads in a metagenomics dataset should be well understood, as there is a risk that the filtered dataset is no longer representative of a real experiment. Experimental data could also be generated using pure cultures of bacteria present as well-characterised strains in biorepositories (see, for example, 46)

These concerns could be addressed by *in silico*-generated datasets, where the exact quantity of reads and genes from each source in the composite dataset can be better controlled. Many tools have been developed for this purpose, simulating reads from the different available platforms (see, for example 47– 51). Once again, it will be important to properly understand these tools, agree on their applicability for the purpose of generating the desired benchmark datasets, and correctly set their parameters so that the resulting simulations are a correct representation of the “real” samples.

***Regarding the quality metrics in the benchmark datasets (e.g. error rate, read quality), should these values be fixed for all datasets, or fall within specific ranges? How wide can/should these ranges be?***

Available published studies of benchmarking NGS bioinformatics pipelines tend to focus on the performance of specific steps at various levels of input quality and/or complexity (SNP rate, GC content, error rate, quality of the reference sequences, contamination, etc.) ^26, 52, 53^. This is different from a fit-for-purpose evaluation of a complete pipeline under conditions where the quality of the input is guaranteed through the application of best practices and quality control of the laboratory component of the procedure. An important consideration is the extent to which the benchmark should challenge the pipeline robustness by including varying levels of, for example, error rates or reads quality. It is likely that a pipeline that works best under optimal conditions would be sensitive to variation of the sequencing run quality. The extent of desired variation should be agreed upon and captured in the *in silico* reference material included in the benchmark.

### 2.2. Samples composition

***How should the benchmark manage the different mechanisms by which bacteria acquire resistance? What is the set of resistance genes/mechanisms that need to be included in the benchmark? How should this set be agreed upon?***

Several mechanisms for the development of resistance to antimicrobials have been characterised ^54^, including: 1) production of an enzyme that digests/metabolizes/modifies the antimicrobial; 2) production of efflux pumps that remove the drug from within the cell; 3) modification, through mutations or biochemical reactions, of the intracellular target of the antimicrobial so that their interaction is lost; 4) activation/upregulation/acquisition of alternate pathways that allow survival by bypassing the pathway disrupted by the antimicrobial; and 5) downregulation of the expression of the pores through which the drug enters the bacteria.

Mechanisms 1), 2) and 4), often involve the acquisition of novel genes by the bacteria from its environment (horizontal transfer) and may be detected, for example, by mapping reads to reference sequence databases that compile such genes. The genetic determinants of mechanisms 3) and 5), however, vary on a case-by-case basis, and may require the detection of Single Nucleotide Polymorphisms (SNPs), insertions/deletions (indels) or variations of copy numbers. These represent different types of bioinformatics determinations which a comprehensive pipeline must be able to resolve, and the benchmark needs to reflect this reality by ensuring that the various types of AMR determinants are correctly represented in the dataset.

Many recent evaluations of the use of NGS for the determination of AMR have emphasised the difficulty of establishing a curated knowledge base of drug resistance genetic determinants to be used as a reference database in NGS data analysis ^2, 13, 55^. The same problem is mirrored in the design of a benchmark that would ensure all determinants are correctly detected. It is also of foremost relevance to consider that certain genetic determinants such as efflux pumps (mechanism 2 above) are notorious for giving false positive results, as they perform a variety of export functions not necessarily related to antibiotic resistance (see, for example, 56). Eliminating these from the search parameters of bioinformatics pipelines was shown to improve positive predictive value ^57^. The results of testing a pipeline using a benchmark dataset involving all mechanisms must be interpreted with the aim of the pipeline in mind, and this should be taken into account when/if criteria are set (see also section 2.3).

Alternatively, choosing to focus a benchmark dataset on specific resistance mechanisms could simplify the task, but these choices would need to be agreed upon, justified and the limitations clearly stated. This reflection is to be linked to ongoing extensive discussions on the generation of appropriate databases of resistance genes and correct interpretation of resistome profiles (see 24, 58). An *a priori* statement can be made that the benchmark dataset should focus on mechanisms of *acquired* bacterial resistance. Similarly, for lack of being exhaustive in terms of the AMR genetic determinants it includes, a set of *in silico* reference materials can be composed of the resistance mechanisms most relevant for public and environmental safety, for example, focusing on certain specific plasmids and AMR genes which have been identified as being important in clinical infections.

The decisions through which specific resistance mechanisms are included in/excluded from the benchmark should be clear, transparent, agreed upon and justified in order to ensure that the benchmark is relevant to the types of risks considered. These will vary depending on the purpose of the experiment (e.g. environmental risk, clinical decision making), and will be important to evaluate whether the same resistance factors can be incorporated into a single benchmark dataset or if different resources will be needed. Transparency is important since these decisions will also guide the inclusion/exclusion of novel resistance mechanisms according to the changing epidemiology over time (see also section 2.4).

***Should datasets representing different sample types (e.g. isolated clones, environmental samples) be included in the same benchmark? Is a correct representation of different bacterial species (host genomes) important?***

The preceding section focused on the nature of the genetic determinants to be included in the *in silico* datasets. These sequences (i.e. AMR genes), however, represent a very small fraction of the overall totality of the sequence data generated from biological materials (e.g. bacterial genomes) in a given experiment. The nature of these majority “background” reads (bacterial host genomes, other contaminants in the sample etc.) in the components of a proper benchmark dataset thus needs to be carefully considered, as they can influence the accuracy of the pipelines.

The detection of drug resistance in clinical settings is often performed by sequencing pure cultured isolates ^18, 59, 60^. Pathogens of particular concern in the context of nosocomial infections will, accordingly, need to be properly represented in the *in silic*o datasets. Lists of AMR pathogens presenting significant risks are maintained (see 61) and include the ESKAPE pathogens ( *Enterococcus faecium*, *Staphylococcus aureus*, *Klebsiella pneumoniae*, *Acinetobacter baumanii*, *Pseudomonas aeruginosa* and *Enterobacter* sp.) and *Escherichia coli*, among others.

Culture-dependent methods cannot be systematically applied to environmental samples for various reasons, including the fact that most environment bacteria are not recovered under standard culture conditions ^62^. Culture-independent approaches (metagenomics) can then be used to analyse the human and environmental resistomes within complex bacterial populations ^13, 25, 63^. These approaches have also been proposed for clinical purposes, greatly reducing the time necessary for characterisation ^8, 16^. For these samples, agreeing on a realistic genetic diversity within a benchmark ^64^ - a set of communities which can be considered “representative” - is a significant challenge as there is tremendous variability in the species composing the microbiomes of different communities ^13, 65– 67^.

### 2.3. Evaluation of pipeline performance

***How can the “true” value of the samples, against which the pipelines will be evaluated, be guaranteed? What is needed to demonstrate that the original sample has been correctly characterised, in case real experiments are used?***

One of the objectives of validating a bioinformatics pipeline is to demonstrate that its accuracy is above an acceptable value, with low instances of false negative and false positive results ^68^. Antimicrobial susceptibility testing using traditional methods is, in itself, a complex procedure subject to differences in methodologies and interpretations ^69^; hence they have required (and will require) validation and standardisation ^70– 72^. There have been reports where discrepancies between NGS-based predictions and susceptibility testing were caused by isolates with inhibition zones close to the susceptibility breakpoint. It was suggested that the results could have been concordant if the susceptibility testing had been performed under different culture conditions, for example, with a different culture medium ^73^. The extent to which these “borderline” cases should be included in the benchmark or not, and the final “correct” prediction that will be attached to them will need to be carefully considered. It should also be discussed what the most relevant endpoint in this context is, between, for example, the Minimum Inhibitory Concentration (MIC) prediction and resistance levels above wildtype/type strain.

The realities of veterinary medicine, with specific modalities of antimicrobial administration, mean that susceptibility MIC breakpoints may differ between humans and animals ^74^. Thus, the definition of science-based clinical MIC-breakpoints (CBPs) is relevant to interpret results and to harmonise the results of antimicrobial susceptibility testing of veterinary pathogens. Currently, this issue is being discussed in different working groups led by VETCAST. This may cause difficulties in assigning a universal “correct” label to some datasets that would apply to both humans and animals.

Reference samples of metagenomics experiments are even more complex in this regard, with each sample containing numerous instances of genetic AMR determinants ^12, 14, 75^. Metagenomics analyses can detect genes (genotype), which are not necessarily translated into resistance (phenotype); expression of the protein(s), which is not directly revealed by DNA sequencing, is important in this context. Assigning accurate profiles to components of a reference dataset will be challenging, as there is no existing pipeline recognised as the ‘gold standard’ to do so ^8^. Spiked samples or simulated reads may be a necessary initial step in this context.

Ultimately, the “true” values to be assigned to the samples in the dataset, and the challenges this will pose, will depend on what the validated pipelines will be required to achieve. For example, the benchmark has to be adaptable to whether the aim is detecting the genetic determinants of AMR, predicting AMR or (for human and veterinary health) predicting antimicrobial susceptibility and thus treatment outcomes.

***How should the target performance thresholds (e.g. specificity, sensitivity, accuracy) for the benchmark suite be set? What is the impact of these targets on the required size of the sample set?***

Validation of a process involves the determination of various performance parameters, such as specificity, sensitivity, accuracy, etc. ^32^. When used specifically for the detection of antimicrobial resistance the benchmark resources need to include strict performance thresholds, and whether these should be set *a priori* along with the levels of these thresholds are subjects for consideration. One also needs to clarify how the process can cope with cases where more than one type of resistance needs to be identified in a single sample, in particular for metagenomics studies.

These performance parameters will be important, not only as information to be included in the benchmark, but also because they generally have a significant influence on the size of the *in silico* dataset needed (see, for example, 76, 77). Understanding the target performance characteristics of a valid pipeline will be necessary to guide decisions as to how many samples will be needed in the *in silico* dataset, with respect to the presence or absence of AMR genetic determinants. Finally, not all parameters are equally important for all samples - for example, considerations of sensitivity are generally not relevant in the case of cultured isolates as the bacteria are present in high numbers, but may be crucial for metagenomics experiments where the proportion of the target(s) relative to the background is variable and unknown. Targeted metagenomics seem promising approaches for the accurate detection of minority genes in complex samples ^13^, and challenging the sensitivity of bioinformatics pipelines with a benchmark dataset would be of added value in this context.

### 2.4. Generation, distribution and update of the benchmark

***How can the benchmark stay relevant when new resistance mechanisms are regularly characterised? How is the continued quality of the benchmark dataset ensured?***

An important fact concerning antimicrobial resistance - and one of the reasons it represents a global health emergency - is that novel mechanisms of resistance are constantly being reported and new genes and/or vectors of transmission regularly emerge ^58, 78^. Assuming that a benchmark resource can be produced covering the existing complexity of AMR determinants (section 2.2), adapting this resource to new information is a challenge that will need to be addressed in order to ensure that its utility does not diminish with time. Criteria for inclusion of new *in silico* datasets, and the mechanisms by which these decisions should be taken, need to be discussed and agreed upon when developing the resource.

Newly identified genetic determinants can also impact the information linked to existing datasets in the benchmark resources. These datasets will need to be re-evaluated in view of new information to ensure that their AMR determinants are properly characterised. As an example, this issue was evidenced in 2015 with the identification of *mcr-1* as a plasmid-borne colistin resistance gene ^79^; re-analysis of existing NGS data from *E.coli* isolates from food, feed and hospitalised patients for the previous years in Denmark revealed previously characterised samples containing this gene ^80, 81^.

***Who should generate the benchmark resource? How can it be efficiently shared?***

Current guidelines and recommendations place the responsibility of validating the bioinformatics pipelines (and ensuring reliability after update of any of its components) with the operator/quality manager of the test facility ^32, 33, 39^. In fact, thus far, many different sets of benchmark materials and resources have been produced for local use or within collaborative endeavours (see 34). Benchmark datasets have also been used to compare different methods or tools ^17, 82, 83^. The extent to which these datasets address the concerns described in this document is the subject of a case-by-case evaluation that may become crucial for a wide implementation of NGS technology for routine and regulatory use. An open and inclusive discussion on the different issues (described here or arising upon more detailed considerations) will be important for the development of a resource that can gain wide acceptance and use.

## Conclusions

The aim of this document is to summarise a list of challenges that were identified at the meeting organised by the Joint Research Centre on the 4 ^th^ and 5 ^th^ of October 2017 for the creation of a benchmark resource. The specific objective of this benchmark would be to challenge the bioinformatics step of a workflow to identify antimicrobial resistance in samples, using NGS technologies. It is clear that this covers only a fraction of the work necessary to fully implement this technology in a regulatory context, which will also need to cover additional steps such as the sampling, library preparation, sequencing run, and interpretation of the AMR profiles (see Figure 1). However, this resource would facilitate the implementation of the NGS technology in routine laboratory analyses by:

- Ensuring confidence in the implementation of the bioinformatics component of the procedure, a step currently identified as limiting in the field ^2, 8– 10^.
- Allowing evaluation and comparisons of new/existing bioinformatics strategies, resources and tools.
- Contributing to the validation of specific pipelines and the proficiency testing of testing facilities.
- "Future-proofing" bioinformatics pipelines to updates and replacement of the tools and resources used in their different steps.

Some of the challenges in building such a resource are common to all NGS-based methods. Many reports on standardisation, quality management and good laboratory practice have focused on clinical testing and the detection of germline sequence variants linked to cancer or other diseases and could guide some of the decisions to be taken. In this context, reference materials were highlighted as necessary for test validation, QC procedures and proficiency testing ^68^. However, many of the challenges also reflect the reality of antimicrobial resistance monitoring and are specific to this framework. How much of the available resources can be directly applied or used to guide future efforts in this field will need evaluation and, eventually, complementation.

While the present discussion focuses on the monitoring of bacteria, most of the challenges described herein and the means by which they will be approached should apply to AMR monitoring in other organisms, such as viruses, parasites, and fungi. The differences will be in the final solutions proposed for the composition of the benchmark dataset, due, of course, to the different biology in the mode of action of the antimicrobials and their associated resistance mechanisms.

As it was made apparent in the previous sections, many of the challenges are due to the large heterogeneity behind the reality of detecting AMR using NGS. Some of this heterogeneity will require the development of separate benchmark datasets (e.g. the different sequencing platforms) while some will obviously gain by being combined into a single resource (e.g. human and veterinary medicine). Other cases will require more discussions and evaluations of feasibility/added value in being considered together *vs* separately (e.g. samples composed of isolates *vs* metagenomics).

Whatever the final composition and number of the benchmark resource(s), the proper path will ensure a holistic view of the problem that also reflects current public health data. This decision-making process should include expertise in AMR characterisation in humans, animals, food and the environment, in order to maximise its impact on the establishment of an AMR surveillance framework that is in line with the principles of a “One Health” approach.

## Disclaimer

The contents of this article are the views of the authors and do not necessarily represent an official position of the European Commission.

## Data availability

No data is associated with this article.

## Footnotes

^1^See, for example, https://www.accessdata.fda.gov/scripts/cdrh/cfdocs/cfRL/rl.cfm?lid=427645
