# Supplementary material for: The challenges of designing a benchmark strategy for bioinformatics pipelines in the identification of antimicrobial resistance determinants using next generation sequencing technologies
Source: F1000Res. 2018 Dec 7;7:ISCB Comm J-459. Originally published 2018 Apr 13. [Version 2] doi: 10.12688/f1000research.14509.2 (PMC6039958; doi:10.12688/f1000research.14509.2)
Supplement: Peer review history

# Peer review: Referee response for version 1

**DOI:** 10.5256/f1000research.15794.r35036

**Published:** 2018-06-29

**Authors:** Jason C. Kwong

**Affiliations:** 1 University of Melbourne, Melbourne, Vic, Australia; 2 Austin Health, Melbourne, Vic, Australia

The opinion article by Alexandre Angers-Loustau and colleagues addresses an increasingly encountered question by clinical and public health microbiology laboratories - what are the considerations in developing a benchmark for validating methods to identify antimicrobial resistance determinants from high-throughput sequencing data? This is an important step in using sequencing data for this purpose, and this article reports back on the discussions by a panel of experts in the field of antimicrobial resistance surveillance.

 In general, the article is well-suited to publication in *F1000Research*, offering insight into an issue of global importance, and I thank the authors for making the discussions from the October 2017 meeting organised by the Joint Research Centre public. It is well written and explores each of the discussion questions with supporting literature. My suggestions for improvement are minor and addressing them is left up to the authors' discretion.

 My overall comment is that while many of the relevant considerations are raised, as a clinician and researcher, I am left with the burden of all these concerns, but no clear direction in how to address the challenges (other than a few comments in the conclusion). I appreciate the purpose of the paper was to summarise the challenges identified at the meeting, but I think this (opinion) article could be enhanced and have a greater impact if more expert opinions and ideas on how to proceed were included.

 Minor comments:

 Introduction (page 4)

 It would be nice to know a little more about the panel of experts - for example, what mix of background or fields did the experts come from e.g. clinical or public health microbiology, bioinformaticians, epidemiologists, veterinary microbiology etc.?

 Section 2.1: real experiments vs simulated read sets (page 5)

 This section highlights some important concerns (e.g. filtering host DNA or patented genetically modified food DNA), but these are more relevant to identification of antimicrobial resistance from metagenomic sequencing data - this is alluded to in the text, but not explicitly stated. Given the other technical challenges with this, I wonder if the discussion should focus more on the "real experimental" data from cultured bacterial organisms – the more likely initial validation before validation proceeds to metagenomic datasets.

 Section 2.2: how should the set of resistance genes/mechanisms be agreed upon (page 6)

 The considerations here are well explored, but I wonder if there could be some mention of variation in benchmark datasets depending on the purpose of resistance gene/mechanism detection. For example, detection of AMR genes from environmental or livestock samples may have a different focus, and thus different benchmark targets to detection of AMR genes in organisms from human samples. Even among human samples, there may be differences in the mechanisms prioritised for detection between testing for clinical treatment and testing for epidemiological surveillance (mcr-1 is a good example).

 Section 2.3: how can the "true" value of the samples be guaranteed (page 7)

 Great section and discussion. Along the lines of the discussion, I am interested to hear the authors' thoughts on whether it is sufficient to validate NGS for predicting AMR against standard susceptibility testing (e.g. MIC), or whether clinical studies evaluating treatment outcomes based on NGS detection are required (i.e. treating based on genotype vs phenotype). My only comment for this section is that perhaps some of the commentary is more directed at validation of NGS for "identifying antimicrobial resistance" rather than "identifying antimicrobial resistance determinants" as stated in the title - two subtly different targets. Perhaps some of the discussion could be clarified here.

 “HTF5 format” (page 4) should actually be “HDF5 format”. 

 MIC should be spelt out at the first usage - minimum inhibitory concentration.

I have read this submission. I believe that I have an appropriate level of expertise to confirm that it is of an acceptable scientific standard.

# Peer review: Referee response for version 1

**DOI:** 10.5256/f1000research.15794.r33450

**Published:** 2018-05-11

**Authors:** Giorgio Palù, Enrico Lavezzo

**Affiliations:** 1 University of Padova, Padova, Italy; 2 Department of Molecular Medicine, University of Padova, Padova, Italy

In this opinion article, Alexandre Angers-Loustau and colleagues address a hot topic in Diagnostic Microbiology regarding the implementation of next generation sequencing platforms for the identification of antimicrobial resistances. In particular, they focus on a specific step of the long pipeline which goes from sample collection to data interpretation and management, namely the bioinformatics analysis of sequencing data. 

 It's been more than 10 years since the introduction of the first NGS instrument, but an adequate standardization and harmonization level of the many different platforms is not yet accomplished.

 The article is very well written; it is concise but covers all the important issues that need to be considered when willing to implement such technologies for the purpose of AMR detection. 

 I only have some minor points for the authors:

1. In section 2.1, page 5, regarding the potential presence of sensitive information (e.g. reads of human origin) when including real experiment datasets in the benchmark. I don't see the problem here. How the availability of anonymous DNA sequence information could compromise the patient's privacy? Of course, patient's identity must be separated from sample's ID, but the de-identification process is a standard procedure and others methods have also been developed for this purpose (see for example this paper from Qamar et al. (2014) ^1^). Please discuss about this.
2. Since this is an opinion article, I would like the authors to 'expose' themselves a little bit more on some points. The challenges are presented by explaining the state-of-the-art, but it is not easy to me to understand authors' thinking about the possible solutions. Obviously they can't have answers for all the challenges, but they could express some personal positions (for example, what about "Who should generate the benchmark resource"? To me, there should be a centralized action aimed, as far as possible, at the definition of a global standard (similar for example to the WHO HPV LabNet Proficiency Studies for Evaluating HPV DNA Typing Methods). What do the authors think about this?
3. Finally, no reference is made to microorganisms other than bacteria, but AMR may be related also to viruses and eukaryotic microorganisms. Are there any difference or peculiarity regarding the bioinformatics analysis of sequencing data coming from them?

We have read this submission. We believe that we have an appropriate level of expertise to confirm that it is of an acceptable scientific standard.

## References

[1] QamarNYangYNadasALiuZSztipanovitsJ: Anonymously Analyzing Clinical Datasets. *arXiv preprint arXiv:1501.05916*.2014;
